# MXene-Modified Fiber-Based Electronic Tongue for Sensitive Detection of Antibiotic Residues in Milk

**DOI:** 10.1021/acsomega.5c09760

**Published:** 2026-01-20

**Authors:** Murilo H. M. Facure, Lingyi Bi, Teng Zhang, Luiza A. Mercante, Yury Gogotsi, Daniel S. Correa

**Affiliations:** † Nanotechnology National Laboratory for Agriculture (LNNA), Embrapa Instrumentação, Sao Carlos, SP 13560-970, Brazil; ‡ PPGQ, Department of Chemistry, Center for Exact Sciences and Technology, Federal University of Sao Carlos (UFSCar), Sao Carlos, SP 13565-905, Brazil; § A. J. Drexel Nanomaterials Institute and Department of Materials Science and Engineering, 6527 Drexel University, Philadelphia, Pennsylvania 19104, United States; ∥ Institute of Chemistry, Federal University of Bahia (UFBA), Salvador, BA 40170-28, Brazil

## Abstract

The widespread use of antibiotics has raised concerns about their residues in dairy products, meat, fish, and poultry, which can pose risks to human health and lead to substantial economic losses. Therefore, the rapid, sensitive, and cost-effective detection of low concentrations of various antibiotics in food samples is critical. This work reports on the fabrication of MXene fibers by coating commercial nylon yarns with Ti_3_C_2_, Ti_3_C_1.75_N_0.25_, and Ti_3_C_1.5_N_0.5_ MXenes and their use as electrodes in an impedimetric electronic tongue (e-tongue). The MXene-modified fiber-based e-tongue was employed in the detection of trace amounts of cloxacillin benzathine, tetracycline hydrochloride, and streptomycin sulfate. By treating the collected electrical resistance data, the system could differentiate the antibiotics and detect their presence in real milk samples at concentrations as low as 10 nM. The use of low-cost MXene-modified nylon fibers as electrodes, which can be fabricated through rapid and straightforward methods, enhances the scalability and practicability of the e-tongue system. This approach represents a promising and robust alternative for the sensitive detection of diverse antibiotic residues in food matrices.

## Graphical abstract

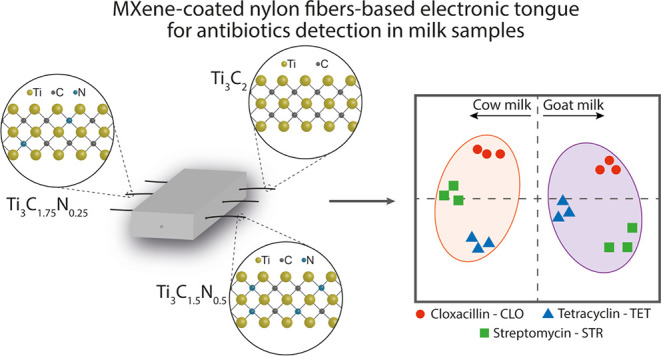

## 1 Introduction

The discovery of penicillin in 1928 marked the beginning of the antibiotics revolution. Since then, these medications have been widely used in human medicine, agriculture, and animal husbandry to treat infections. However, their extensive use has also raised concerns about antibiotic residues in animal-derived food.^1,2^ Moreover, the misuse of antibiotics in livestock can contribute to antimicrobial resistance and trigger allergic reactions, leading to substantial economic losses. Among animal-derived foods, milk and dairy products are particularly affected, making the monitoring of antibiotics’ residues in milk of fundamental importance.^3,4^

The detection of antibiotic residues in milk has been investigated using a variety of analytical techniques, including enzyme-linked immunosorbent assays (ELISA),^5^ optical,^6^ electrochemical,^7^ and chromatographic^8^ methods. Despite the good sensitivity of such methods, which allows for the detection of antibiotics in milk below the maximum residue limit (MRL), i.e., generally around 100 nM,^9^ they have several limitations. In addition to being costly, they typically require complex sample pretreatment, specialized equipment, and trained personnel. As a result, there is a growing need for the development of reliable, sensitive, and cost-effective methods capable of detecting and differentiating antibiotic residues in milk samples.^4,10^

An electronic tongue (e-tongue) is an analytical instrument composed of multiple sensing units.^11,12^ Its characteristic cross-sensitivity arises from the ability of each sensing unit to respond differently to various compounds present in the sample, rather than being selective for a single analyte. Unlike traditional chemical sensors that rely on high molecular selectivity, e-tongues operate based on the concept of global selectivity, in which each sensing unit exhibits partial and overlapping responses to several components of the sample. The collective response of all sensors generates a unique signal pattern or fingerprint for each analyzed sample.^13,14^ With appropriate data processing techniques, these patterns enable the differentiation of samples and the detection of specific analytes.^15,16^

The detection performance of an e-tongue is strongly associated with the choice of materials used to fabricate the sensing units, as they must be responsive to interactions with the analyte under investigation. In this context, the use of nanomaterials in e-tongue systems has led to remarkable improvements in detection performance.^17,18^ Recently, 2D carbides and nitrides, known as MXenes,^19,20^ have been explored as sensing materials for e-tongues^21,22^ and e-noses,^23,24^ achieving promising results. These materials are particularly attractive for sensing applications due to their high electrical conductivity, which enhances signal transduction and improves the signal-to-noise ratio, as well as their large specific surface area and rich surface chemistry.^25,26^ The structure and composition of MXenes, including the presence of surface functional groups, enable multiple interaction pathways with varied analytes, such as adsorption by hydrogen bonding, π–π interactions, or electrostatic effects, which can modulate the electronic properties of the MXenes and generate measurable electrical responses.^25,27^

However, certain issues associated with e-tongues must be addressed to gain commercial interest. One of the primary disadvantages is the need for system recalibration whenever a sensing unit has to be replaced.^15,28^ In addition, the use of expensive materials, such as platinum and gold,^22,29,30^ to fabricate the electrodes of the sensing units increases the products’ cost and hinders their commercial viability.^15^ Furthermore, commercialized sensor arrays are typically large and restricted to use by specialists in limited places.^31^ Therefore, the development of compact and cost-effective sensing units that can be easily and rapidly fabricated is highly sought after.

Here, we report the fabrication of MXene-coated nylon fibers using titanium carbide and carbonitride compositions (Ti_3_C_2_T_*x*_, Ti_3_C_1.75_N_0.25_T_*x*_, and Ti_3_C_1.5_N_0.5_T_*x*_, where T_*x*_ stands for surface terminations) as low-cost electrodes for an impedimetric e-tongue, as illustrated in Scheme 1a. These MXenes were selected because variations in their carbonitride structures and surface terminations give rise to distinct electrical behaviors, enabling the generation of complementary sensing responses for the e-tongue. Additionally, their inherently high electrical conductivity makes them particularly suitable as sensing elements in devices operating by electrical impedance. In this context, MXenes were employed to coat nylon fibers to produce sensitive, low-cost, and straightforward-to-fabricate electrodes. These modified electrodes were used in an e-tongue system to detect and differentiate trace amounts of three antibiotics, namely cloxacillin (CLO), tetracycline (TET), and streptomycin (STR), in cow and goat milk samples (Scheme 1b).

**Scheme 1:**
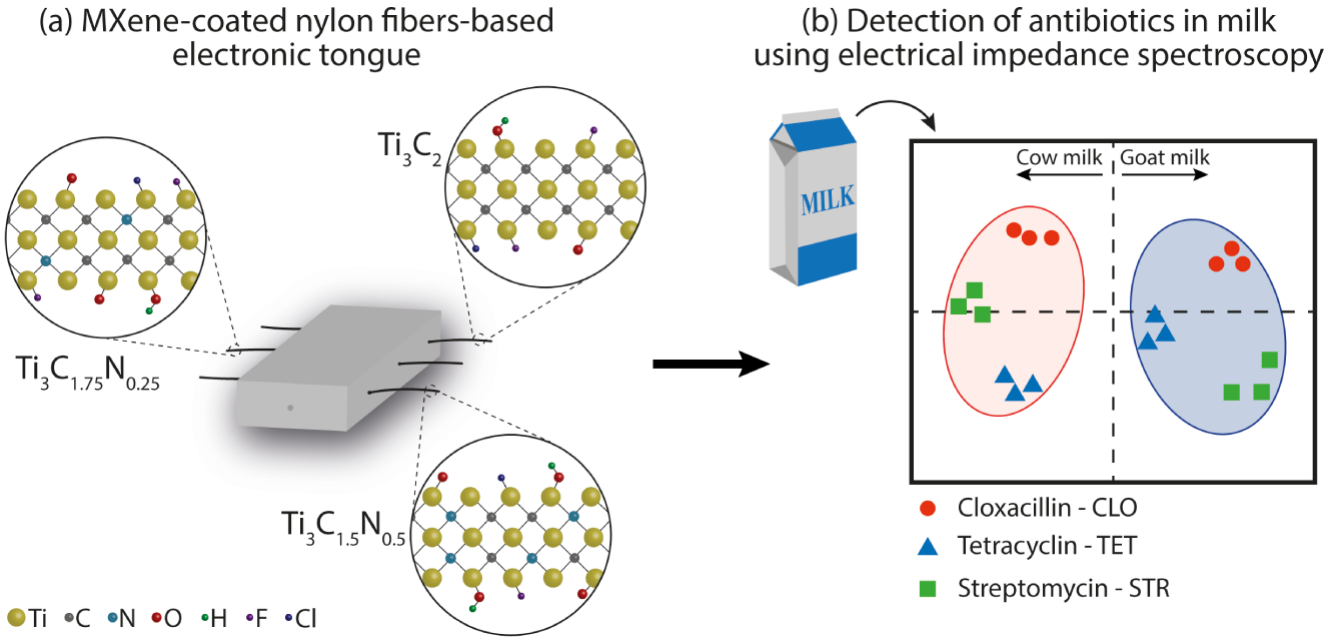
Schematic Illustration of the (a) Electronic Tongue (Using Impedance Spectroscopy) Composed of Sensing Units of Nylon Fibers Coated with Different Titanium Carbonitride Compositions and Their Surface Terminations; (b) Representation of Antibiotic Detection in Cow and Goat Milk Using Principal Component Analysis (PCA) of Experimental Data Collected with the MXene-Modified Fiber-Based Electronic Tongue

## 2 Materials and Methods

### 2.1 Materials

Elemental Ti (Alfa Aesar, −325 mesh, 99.5%), Al (Alfa Aesar, −325 mesh, 99.5%), AlN (Fisher Scientific, −325 mesh, *N* ≥ 32.0%), and graphite (Alfa Aesar, −325 mesh, 99%) powders were used to obtain the MAX phases. Hydrochloric acid (HCl, Fisher Scientific, 38 wt %), hydrofluoric acid (HF, 50%, Acros Organics), and lithium chloride (LiCl, 99%, Acros Organics) were used in the synthesis and delamination of MXenes.

Continuous nylon filaments with a diameter of 300 μm and a round cross-section were purchased on spools from Thread Exchange Inc.

Cloxacillin benzathine (CLO), tetracycline hydrochloride (TET), streptomycin sulfate (STR) (Figure S1), monopotassium phosphate (KH_2_PO_4_), and dibasic potassium phosphate (K_2_HPO_4_) were obtained from Sigma-Aldrich. Ethanol (≥99.0%) was purchased from Vetec. Commercial goat and cow milk used in the real sample analysis were purchased in a local market.

### 2.2 MXene Synthesis and Production of the MXene-Coated Fibers

Ti_3_AlC_2–*y*_N_*y*_ (where *y* = 0, 0.25, or 0.5) MAX phases were synthesized based on our previous report.^32^ For each composition, Ti, Al, AlN, and C powders were mixed in a molar ratio of 3:1:2 (M:A:X basis). In the process, 50 g of MAX phase powder was first ball-milled for 16 h in a polypropylene milling jar. After milling, the powders were passivated in ambient air overnight. Then, the passivated powders were heated at a rate of 3 °C/min to 1550 °C and reacted for 6 h in a high-temperature tube furnace (MTI). The furnace tube was purged with ultrahigh-purity Ar (200 sccm) for 1 h before synthesis. The resulting lightly sintered blocks were ground using a mortar and pestle to obtain fine powders. To remove intermetallic impurities, the powders were stirred in HCl overnight, followed by repeated washing with deionized water until neutral pH. The cleaned powders were dried under vacuum at room temperature overnight and subsequently sieved through a stainless-steel mesh (<38 μm) for further use.

The MXenes were synthesized by selectively etching the Al layer of the MAX phase. For 1 g of Ti_3_AlC_2–*y*_N_*y*_ MAX phase, a mixture of 2 mL of HF, 6 mL of water, and 12 mL of 12 M HCl was used as etchant. After adding the MAX phase powder to the acidic solution, the mixture was stirred at 350 rpm and 35 °C for 24 h. Then, the synthesized multilayer MXene was washed until neutral pH using centrifuge cycles of 3500 rpm for 5 min. The delamination was performed by mixing 1 g of the multilayer MXene with 20 mL of a 1 M LiCl solution, which was stirred for 24 h for intercalation. Then, delaminated MXene was obtained by collecting the dark supernatant obtained after centrifuging the mixture at 3500 rpm for 10 min. MXene produced by this method exhibit O/OH surface terminations with some amounts of fluorine and chlorine (Figure S2). However, for simplicity, we will not add T_*x*_ to the chemical formulas of MXenes in the article and refer readers to the XPS data in SI that show the chemical composition of the produced MXenes (Figures S2 and S3).

MXene dispersions with varying C–N ratios were initially concentrated through high-speed centrifugation (10,000 rpm, 20 min). Their concentrations were calculated by weighing free-standing MXene films obtained from filtering dispersions containing 1 mL of the concentrated MXene dispersion. The MXene dispersions were then adjusted to a concentration of 30 mg/mL using DI water.

Nylon filaments were coated with the MXene dispersions following the procedures reported by Bi et al.^33^ The fiber coating process was performed by threading the loose end of a nylon filament through a needle tip and inserting it into a tube containing the MXene dispersion. Then, the loose end of the filament was pulled up at a controlled speed (15 mm/s) using a universal tension machine, depositing a thin MXene layer onto the nylon fiber surface. Multiple nylon filaments of approximately 70 cm in length were coated with each MXene composition and later used for characterization and electrical measurements. The positive charge on the surface of nylon leads to good adhesion of negatively charged MXene flakes to the fiber surface, preventing debonding of the coating. Digital pictures of a fiber used in this work and the developed e-tongue device based on the MXene-coated fibers are shown in Figure S4.

### 2.3 Physicochemical Characterization

Scanning Electron Microscopy (SEM) images of the MXene-coated fibers were obtained using a JEOL JSM-6510. X-ray photoelectron spectroscopy (XPS) analyses were carried out using a PHI VersaProbe 5000 (Physical Electronics) equipment with a 100 μm spot size and a 25 W monochromatic Al K_α_ (1486.6 eV) X-ray source. The binding energy scale was calibrated using the Ti–C peak of C 1s at 282.0 eV. A Tougaard background was applied for transition-metal-containing spectra.

### 2.4 Antibiotic Solutions

Antibiotic stock solutions (1 mM) of CLO, TET, and STR were prepared by dispersing the respective antibiotic powders in phosphate buffer solution (PBS, pH = 6.7, 0.1 M) containing 5% (v/v) of methanol. The solutions used in the electrical measurements were obtained by diluting the stock solution with PBS at 10 nM, 10 μM, and 100 μM, and were immediately used in the experiments. The chemical structures of the antibiotics analyzed in this work are presented in Figure S1.

To evaluate the feasibility and practical application performance of the e-tongue, cow and goat milks were used as real samples. The milk samples were filtered with a 0.22 μm syringe filter to remove large particles, diluted 10-fold, and spiked with various concentrations of the antibiotics.

### 2.5 Impedance Spectroscopy Measurements

To perform the impedance spectroscopy measurements, a Solartron impedance analyzer (1260 A) was used. Fiber pieces with 3 cm lengths were cut, and electrical contacts separated by 1 cm were used to make the measurements. Ten μL of solution was dropped onto the fiber located between the contacts, and after a stabilization time of 2 min, the resistance data were recorded at an applied voltage of 100 mV, with frequency ranging from 1 MHz to 1 Hz, collecting 5 points per decade. All measurements were performed in triplicate.

### 2.6 Data Treatment

The recorded electrical resistance data were analyzed using Principal Component Analysis (PCA). PCA uses a mathematical algorithm to reduce the dimensionality of the experimental data while retaining most of the variation, yielding directions called “principal components” (PCs). Each PC is uncorrelated with the others. The first principal component (PC1) denotes the axis along which the data set exhibits the maximum variance. The second principal component (PC2) captures the second-highest variance and remains orthogonal to PC1, thereby ensuring no correlation between the two components. The generated PCA graph, obtained by plotting the main PCs, is used to assess whether the samples can be meaningfully grouped based on their similarities and differences, where similarity is indicated by proximity and dissimilarity by greater distances.^31,32^

## 3 Results and Discussion

### 3.1 Materials Characterization

The morphology of the MXene-coated fibers was evaluated by SEM images (Figure 1). According to Figure 1a,c, and d, all MXenes formed a uniform coverage on the nylon fiber, without macrocracks or breaks. The uniform MXene coating with strong adhesion is essential for preventing material detachment, ensuring the mechanical stability of the fibers during handling, and enabling reproducible electrical measurements. The SEM images at higher magnifications (Figure 1b,d, and f) show the features of the fiber surface, suggesting a very thin and conformal coating, and a slightly wrinkled surface of the coating formed during drying of the MXene, especially Ti_3_C_1.5_N_0.5_. A wrinkled surface can be beneficial for sensor sensitivity, since it increases the surface area available for interaction with the analyte under investigation.^34^

**Figure 1:**
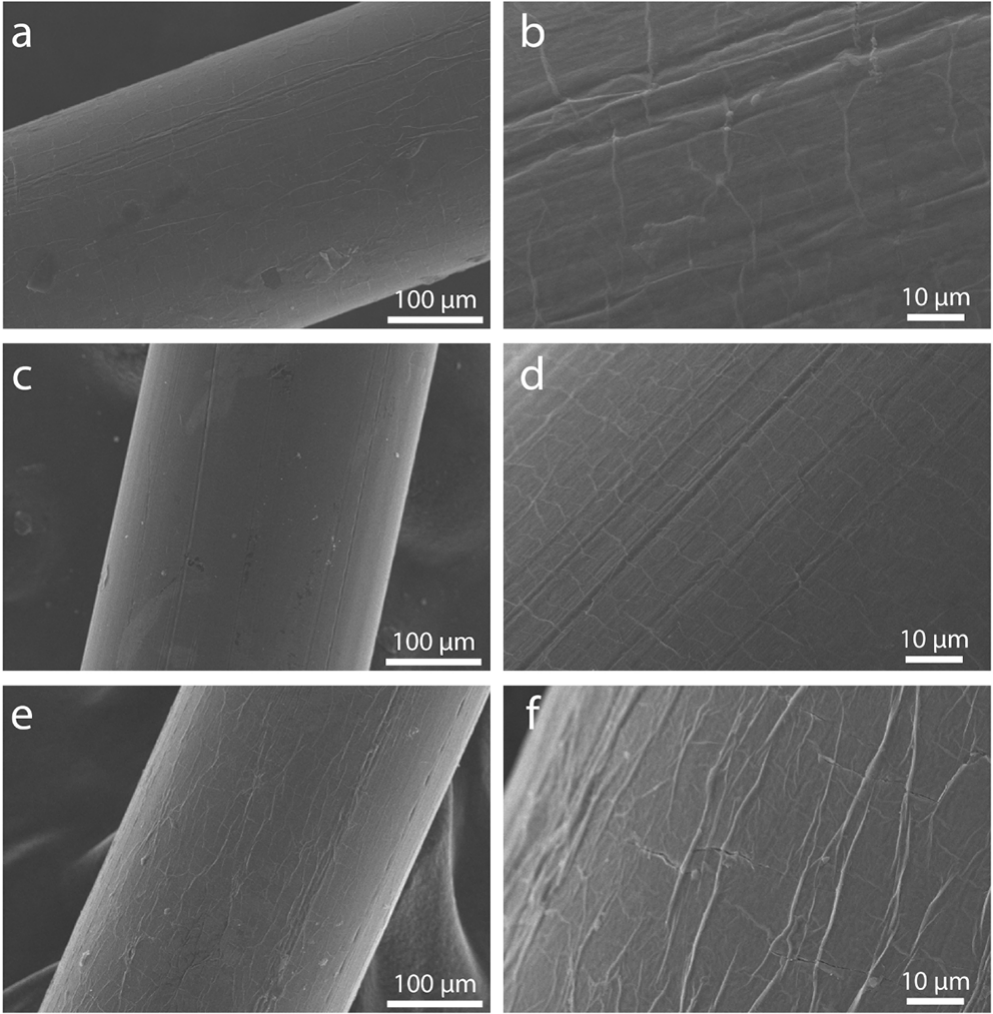
SEM images of MXene-coated nylon fibers using (a) and (b) Ti_3_C_2_, (c) and (d) Ti_3_C_1.75_N_0.25_, and (e) and (f) Ti_3_C_1.5_N_0.5_.

The XPS survey spectra of the synthesized MXenes (Figure S2) show that while the N 1s peak is absent from the Ti_3_C_2_ spectrum, it appears in the Ti_3_C_1.75_N_0.25_ and Ti_3_C_1.5_N_0.5_ spectra, confirming the presence of N in these MXenes. As shown in Figure 2a, the high-resolution C 1s spectra can be divided into four singlet peaks, corresponding to C–Ti, C–C, C–O, and C=O bonds.^35^ The decrease in the C–Ti peak intensity as the amount of N in the MXene formula increases indicates the substitution of C by N. Additionally, the intensity of the peak assigned to C=O increases with N content, which can be attributed to changes in the proportions of surface termination groups (T_*x*_) among the different materials. This trend suggests an increase in O content as the N concentration increases. Indeed, by fitting the relative intensities of the O 1s, F 1s, and Cl 2p peaks, the O-containing surface terminations increase with N concentration on the MXene structure, while the F terminations percentage reduces (Figure S3). The peaks’ positions of the C 1s spectra and their respective integrated areas are shown in Table S1.

**Figure 2:**
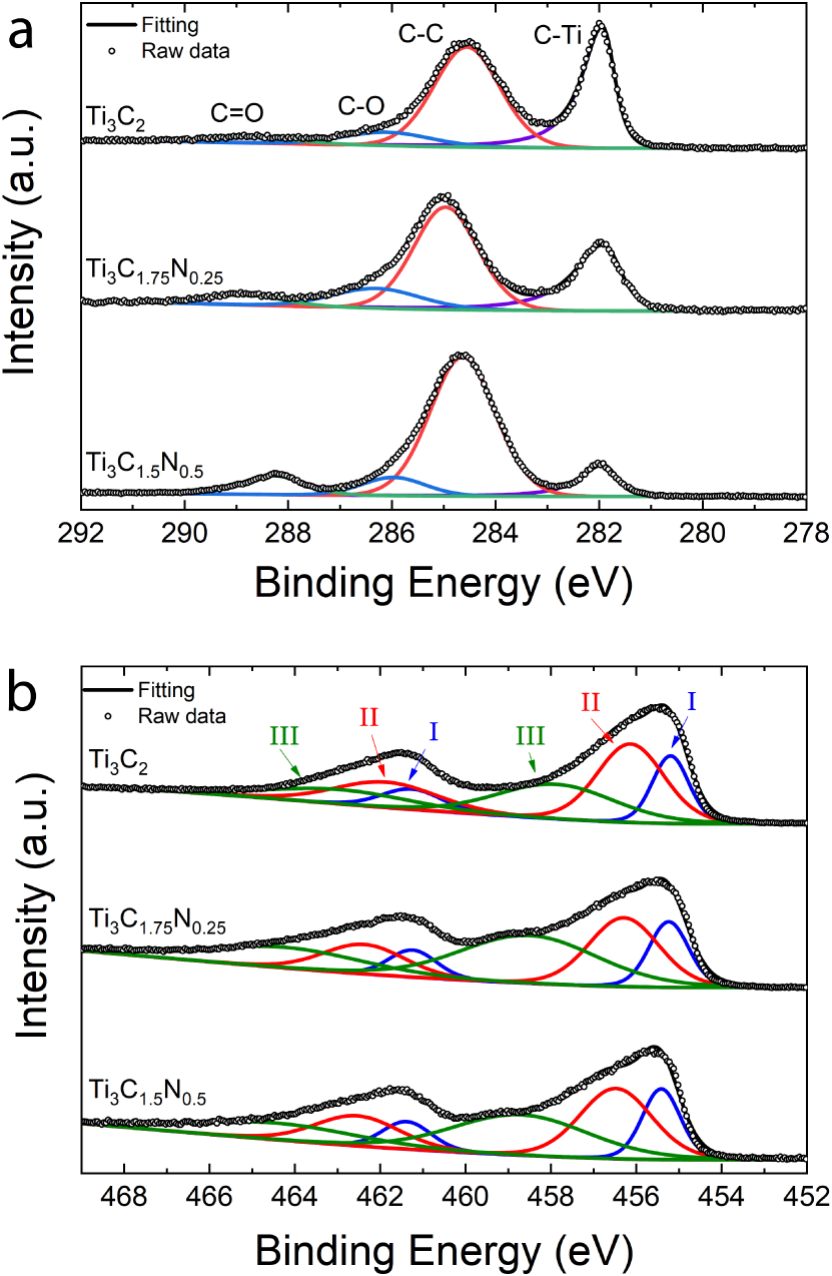
High-resolution XPS spectra of (a) C 1s and (b) Ti 2p of Ti_3_C_2_, Ti_3_C_1.75_N_0.25_, and Ti_3_C_1.5_N_0.5_ MXenes used to coat the nylon fibers.

Figure 2b shows the Ti 2p high-resolution spectra that are fitted by three doublets corresponding to Ti 2p (3/2) and Ti 2p (1/2) with a fixed ratio of 2:1. Peak I is ascribed to the inner Ti atoms (Ti–C), peak II refers to the outer Ti (Ti^2+^), and peak III is related to high-valence-state Ti, including Ti^3+^, Ti atoms bonded to X and F atoms, and TiO_2_ (Ti^4+^). For Ti_3_C_2_, peak III can be mainly associated with the surface Ti atoms bonded to oxygen, while for Ti_3_C_1.75_N_0.25_ and Ti_3_C_1.5_N_0.5_, it is related to the Ti atoms bonded to nitrogen in the X-layer.^36^ Due to the incorporation of N in the MXene lattice, the Ti oxidation state increases, as reported previously.^35,36^ The positions and the integrated areas of the deconvoluted peaks of Ti 2p spectra are presented in Table S2.

Figure S5 shows the N 1s spectra of Ti_3_C_1.75_N_0.25_ and Ti_3_C_1.5_N_0.5_. The peaks centered at 396.8 and 397.2 eV can be assigned to N at the lattice site.^36^ The XPS analysis confirmed the MXene synthesis and revealed that increasing the N concentration in the MXene formula leads to changes in surface terminations and in the Ti oxidation state, which are beneficial for attaining different electrical behaviors.

### 3.2 Electrical Characterization and Antibiotic Detection

Before performing the measurements with the antibiotic-containing solutions, the MXene fibers were electrically characterized by impedance spectroscopy in PBS. The electrical resistance values (Figure 3a) of the fibers increase with the increasing amount of N in the MXene structure and, consequently, with the reduction in the amount of C.^37^ This behavior can be ascribed to changes in the oxidation state of Ti arising from the introduction of N atoms into the MXene lattice and the different concentrations of surface terminations.^36^ Furthermore, variations in the ratio of surface terminations due to the increasing nitrogen content in the MXene structure, as shown in Figure S3, may also lead to significant changes in the electrical properties of the materials.^38–40^ It is also possible to observe that for the fibers coated with MXene Ti_3_C_2_ and Ti_3_C_1.75_N_0.25_, the electrical resistance values exhibit minimal variation across the frequency range. However, for the fiber coated with Ti_3_C_1.5_N_0.5_, the electrical resistance increases in the frequency range from 100 to 1 Hz. The low standard deviations observed corroborate the stability of the fiber during measurements. The different electrical resistance values and response patterns are crucial for the performance of an e-tongue sensor array, as they contribute to the characteristic cross-sensitivity and, consequently, the necessary global selectivity.^16^

**Figure 3:**
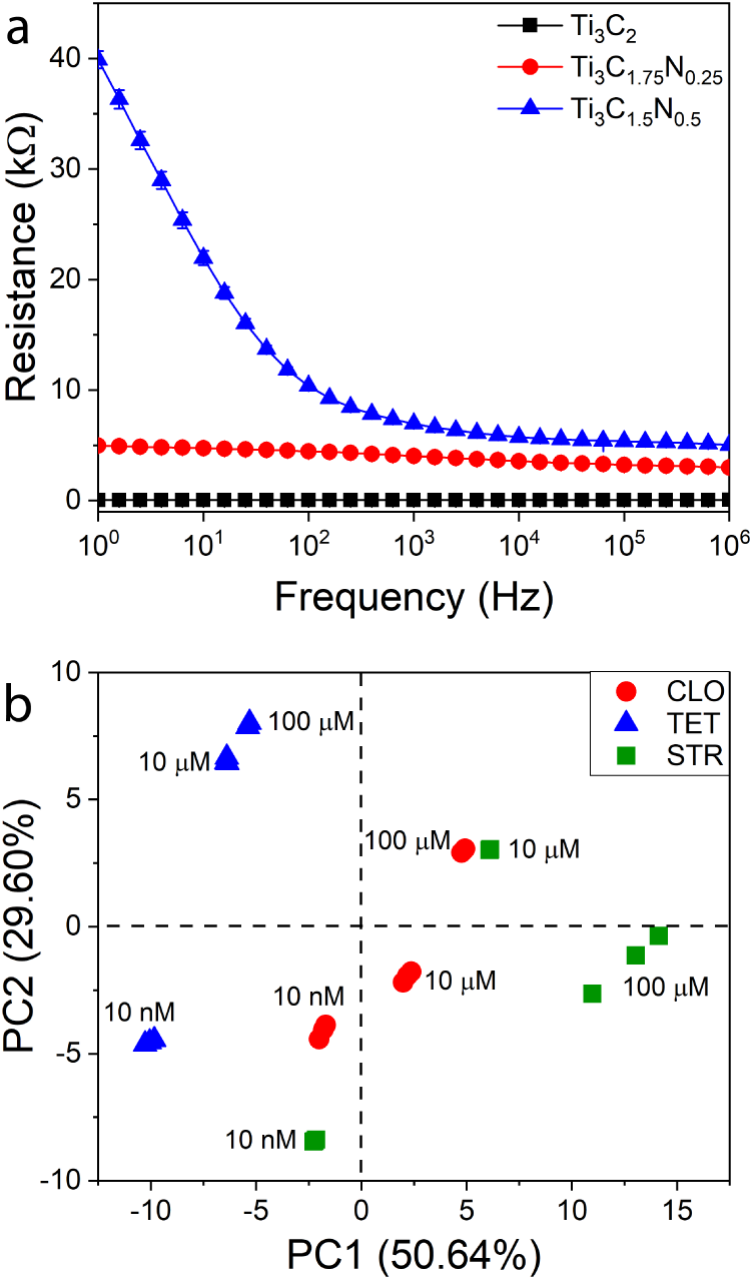
(a) Electrical resistance versus frequency for Ti_3_C_2_, Ti_3_C_1.75_N_0.25_, and Ti_3_C_1.5_N_0.5_ MXene nylon fibers in PBS. (b) PCA plot for the electrical resistance collected by the MXene-coated nylon fibers-based e-tongue in the range from 1 MHz to 1 Hz for the analysis of cloxacillin (CLO), tetracycline (TET), and streptomycin (STR) at different concentrations in PBS. Measurements were performed in triplicate.

To investigate the e-tongue’s capability to distinguish different antibiotics, measurements were initially conducted using PBS solutions containing the target analytes (CLO, TET, and STR). Three concentrations (10 nM, 10 μM, and 100 μM) were evaluated for each antibiotic tested. The PCA plot (Figure 3b) shows that the e-tongue successfully differentiated among the antibiotic types and their concentrations. A correlation is observed between the concentration of the antibiotic solutions and their position on the graph relative to the PC1 value: higher concentrations of antibiotic solutions correspond to higher PC1 values. The proximity of the points corresponding to the same sample indicates good reproducibility of the measurements. Also, a good data correlation is obtained since PC1 + PC2 accounts for more than 80% of the total variance collected by the sensor array. The electrical resistance data collected by each sensing unit during the analysis of antibiotic solutions in PBS and used to obtain the PCA graph shown in Figure 3b are provided in Figure S6.

The ability of the e-tongue to distinguish between different antibiotics is due to variations in the electrical properties of the MXenes and their surface terminations. As a result, the interaction with the distinct analytes generates a specific electrical response for each MXene. Moreover, the presence of different concentrations of surface terminations leads to variations in the interaction of the sensing unit with the investigated analytes.^41^

### 3.3 Real Sample Analyses

To assess the practical applicability of the e-tongue, cow and goat milk samples were used as matrices for antibiotic detection. These samples were chosen to evaluate the e-tongue’s detection performance in matrices with distinct compositions where antibiotic contamination has previously been reported.^7,42^ The electrical resistance data collected from the antibiotic analyses in cows’ and goats’ milk are shown in Figures S7 and S8, respectively. The resulting PCA plots (Figure 4) demonstrate that the e-tongue system can also recognize and distinguish different antibiotics in more complex media, even in the presence of a variety of other organic molecules in milk. In Figure 4a and b, the samples without antibiotic addition are located in the upper-left part of the PCA graph, illustrating the e-tongue’s ability to detect the presence of antibiotics in actual milk samples. Additionally, the separation of samples corresponding to different antibiotics in the PCA plots indicates that the e-tongue can effectively distinguish the type of antibiotic present in the milk sample. Although the tests were performed with samples contaminated with known antibiotics, the good discrimination observed in the obtained PCA graph suggests that the e-tongue can distinguish other antibiotics in real milk samples, opening the possibility of blind tests in future studies.

**Figure 4:**
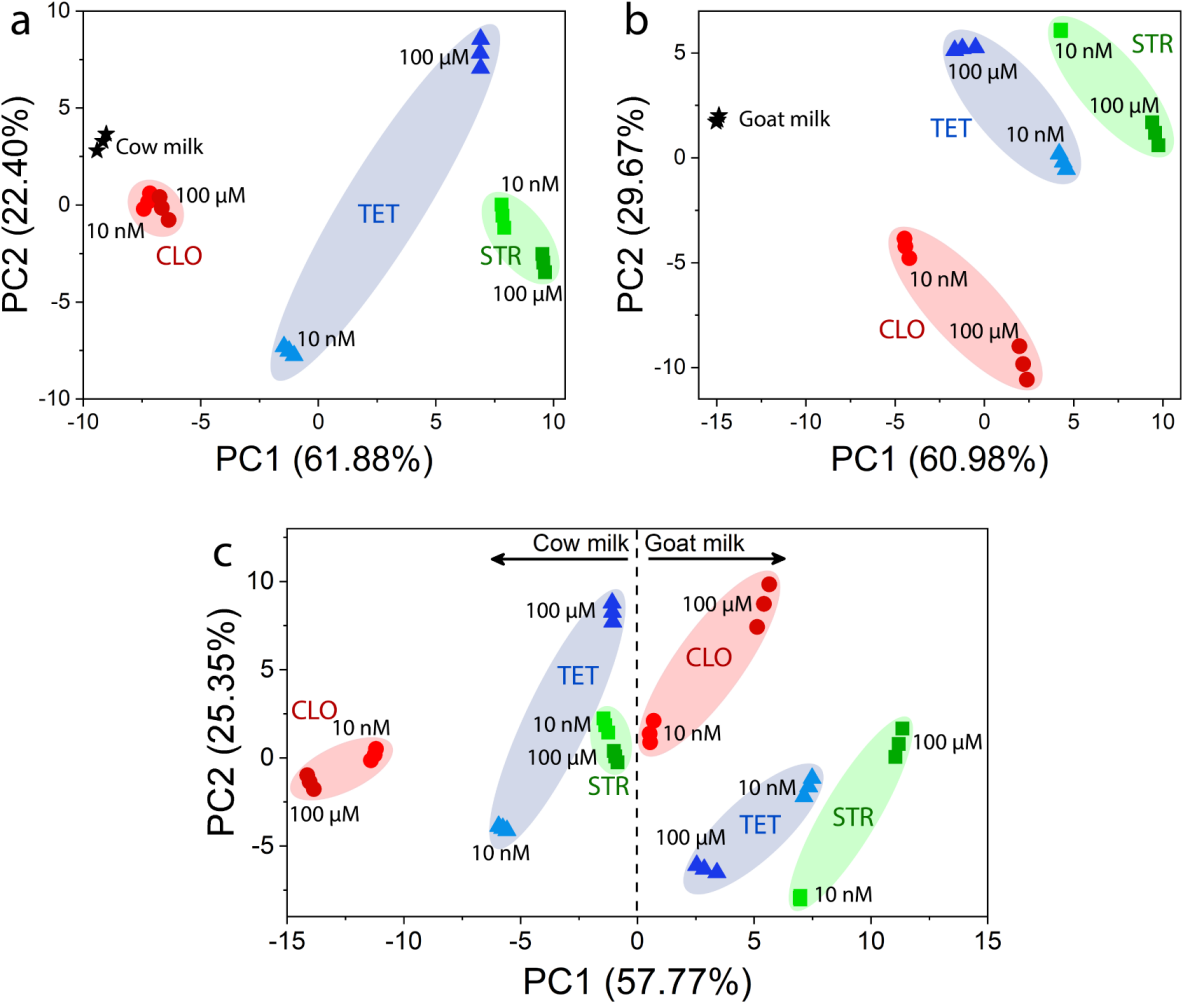
PCA plots for the electrical resistance collected by the MXene-coated nylon fibers-based e-tongue in the range from 1 MHz to 1 Hz for the analysis of CLO, TET, and STR at different concentrations in (a) cow and (b) goat milk samples. (c) PCA plot obtained by treating both data sets together.

When treating the electrical resistance data from cow and goat milk measurements together (Figure 4c), the system successfully distinguishes between cow milk (located in the negative PC1 region) and goat milk (located in the positive PC1 region), while maintaining good discrimination of the antibiotic types and their concentrations. It is important to note that intrinsic matrix components such as fat, proteins, and lactose contribute to the overall impedance response. However, these contributions are incorporated into the global multivariate fingerprint rather than treated as interfering effects, validating the use of the e-tongue for real-sample analysis. Measurements carried out in PBS, as well as in goat and cow milk samples, demonstrate that the e-tongue effectively detected the presence of antibiotics across different media, including complex and compositionally similar matrices such as the milk samples utilized in this study. The e-tongue not only distinguished the concentration and type of antibiotic but also successfully identified the type of milk in which they were present.

Tests were also conducted with mixtures of antibiotics. In Figure S9, it can be observed that the samples without antibiotic contamination are again isolated at the negative PC1 and positive PC2 regions. Additionally, the samples containing all three antibiotics are positioned at higher PC1 values in both graphs, suggesting a trend in the PCA distribution based on the number of different antibiotics present. This pattern is particularly notable, given that all solutions have the same antibiotic concentration (100 μM). When the data are treated including the solutions containing only one antibiotic (Figure 5), a diagonal trend can be observed (toward positive values of PC1 and PC2) as the number of antibiotics present in the solution increases. The electrical resistance data collected from the analysis of solutions containing antibiotic mixtures in cow milk and goat milk are presented in Figures S10 and S11, respectively.

**Figure 5:**
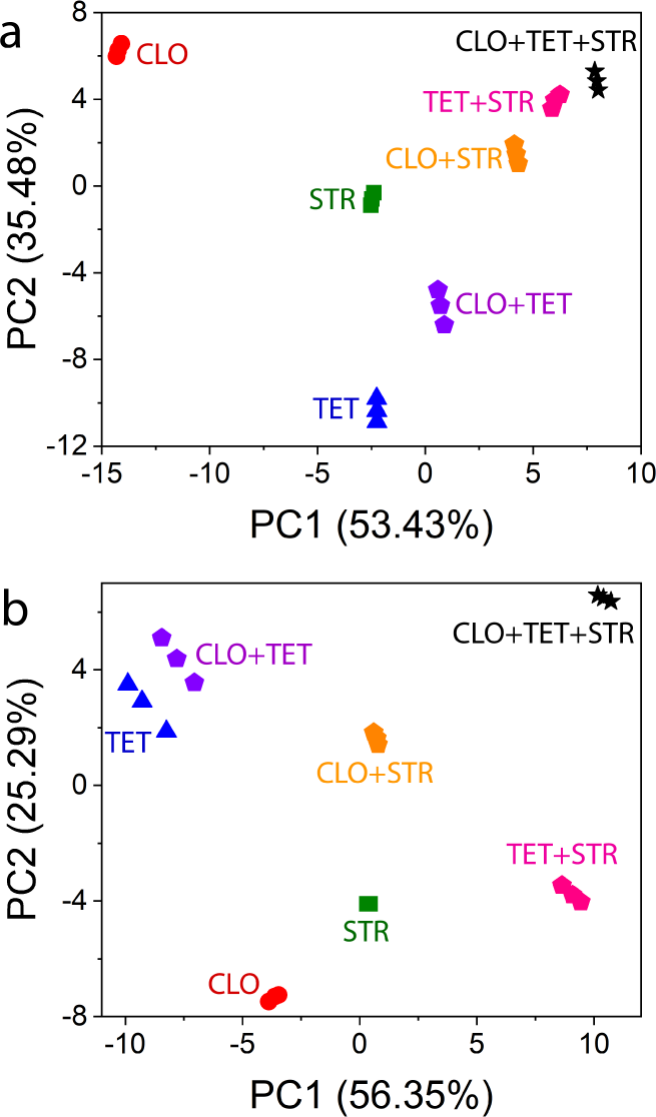
PCA plots for the electrical resistance collected by the MXene-coated nylon fibers-based e-tongue in the range from 1 MHz to 1 Hz for the analysis of solutions containing different numbers of antibiotics CLO, TET, and STR at 100 μM in (a) cow and (b) goat milk.

The results obtained using actual milk samples demonstrate that the e-tongue can be used to distinguish the presence of distinct antibiotics in milk and reveal the potential of the system for real-situation analysis with good accuracy. Considering that over 100 binary and solid-solution MXenes (not counting various surface terminations) have been reported,^43^ one can greatly extend the e-tongue sensitivity and the number of molecules to be detected by using various MXene compositions. One can also vary the diameter and cross-section of the fibers used as substrates for MXene sensors, further tuning the e-tongue performance. It is also important to highlight that, despite the good reproducibility of measurements obtained with the same electrode set, given the low-cost fabrication process of the MXene-coated fibers, the proposed e-tongue could also be used for single-use applications as well.

## 4 Conclusions

MXene-coated nylon fibers were utilized as sensing units of an impedimetric e-tongue designed to detect low concentrations of antibiotics. The distinct electrical properties of the titanium carbonitrides resulted in a sensor array with cross-sensitivity to different antibiotics. The e-tongue successfully distinguished the antibiotics and identified them in both cow and goat milk samples. In addition to differentiating between contaminated and uncontaminated milk, the system also detected the presence of multiple analytes in milk. These results demonstrate the potential of MXene-coated fibers as sensitive and low-cost electrodes for impedimetric e-tongues, offering a promising and economically viable solution for monitoring antibiotic residues in foodstuffs below the maximum residue limit allowed, while addressing key limitations of current detection methods and improving commercial viability. Furthermore, the multivariate fingerprints reported here open the possibility for future studies to investigate blind-test classification of unknown samples using this MXene-based sensing platform.

## Supplementary Material



## Notes

Chemical structures of cloxacillin, tetracycline, and streptomycin, XPS spectra of the Ti_3_C_2_, Ti_3_C_1.75_N_0.25_, and Ti_3_C_1.5_N_0.5_ MXenes, atomic ratio of the MXenes’ surface terminations, pictures of a MXene fiber and the electronic tongue device, tables with the C 1s and Ti 2p XPS data of the MXenes, N 1s spectra of Ti_3_C_1.75_N_0.25_ and Ti_3_C_1.5_N_0.5_, electrical resistance data collected by the MXene e-tongue in the analyses of the antibiotic solutions in PBS, cow and goat milk, and mixtures of antibiotic in real samples, PCA plots for antibiotic mixtures (PDF)
