# Supplementary material for: MXene-Modified Fiber-Based Electronic Tongue for Sensitive Detection of Antibiotic Residues in Milk
Source: ACS Omega. 2026 Jan 20;11(4):5779–86. doi: 10.1021/acsomega.5c09760 (PMC12878441; doi:10.1021/acsomega.5c09760)
Supplement: Supplementary file 1 [file ao5c09760_si_001.pdf]

Supporting Information

MXene-modified fiber-based electronic tongue for sensitive detection of antibiotic residues in milk

Murilo H. M. Facure,[1,2] Lingyi Bi,[3] Teng Zhang,[3] Luiza A. Mercante,[4] Yury Gogotsi[3*], Daniel S. Correa,[1,2*]

[1]*Nanotechnology National Laboratory for Agriculture (LNNA), Embrapa Instrumentação, 13560-970, Sao Carlos, SP, Brazil*

[2]*PPGQ, Department of Chemistry, Center for Exact Sciences and Technology, Federal University of Sao Carlos (UFSCar), 13565-905, Sao Carlos, SP, Brazil*

[3]*A. J. Drexel Nanomaterials Institute and Department of Materials Science and Engineering, Drexel University, Philadelphia, PA 19104, USA*

[4]*Institute of Chemistry, Federal University of Bahia (UFBA), 40170-280, Salvador, BA, Brazil*

* Corresponding authors:
daniel.correa@embrapa.br (Daniel S. Correa)
gogotsi@drexel.edu (Yury Gogotsi)

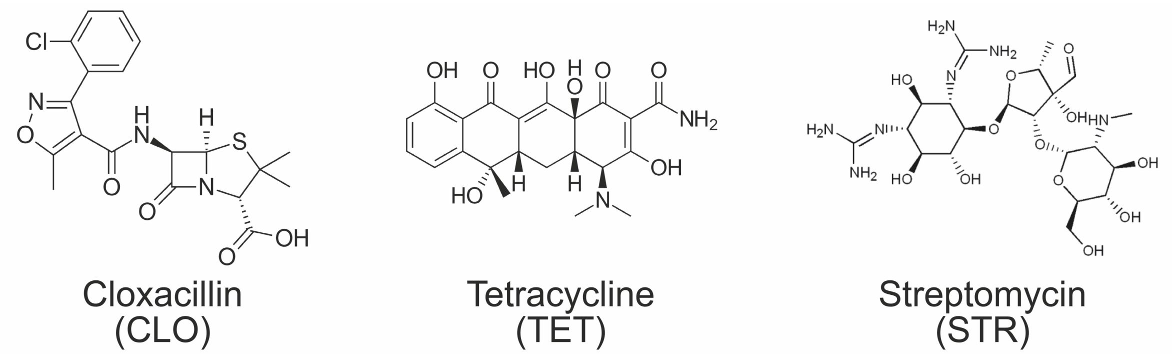

Figure S1. Chemical structures of the antibiotics analyzed: cloxacillin benzathine (CLO), tetracycline hydrochloride (TET), and streptomycin sulfate (STR).

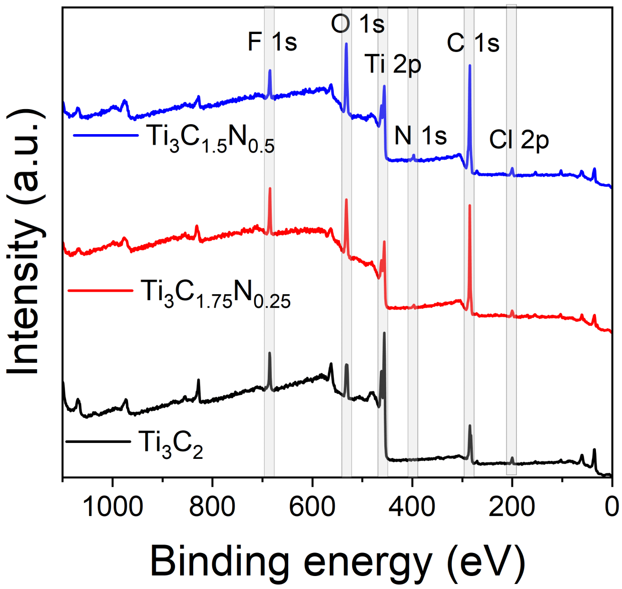

Figure S2. XPS survey spectra of Ti_3C_2, $Ti_3C_{1.75}N_{0.25}$, and $Ti_3C_{1.5}N_{0.5}$ MXenes.

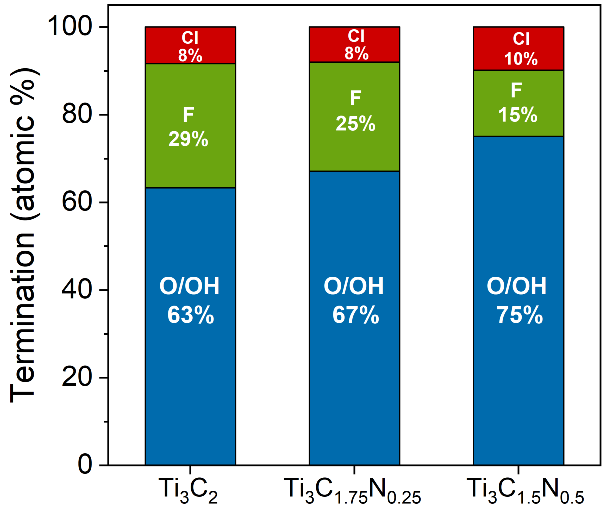

Figure S3. Termination ratio in atomic percentage of the Ti_3C_2, $Ti_3C_{1.75}N_{0.25}$, and $Ti_3C_{1.5}N_{0.5}$ MXenes.

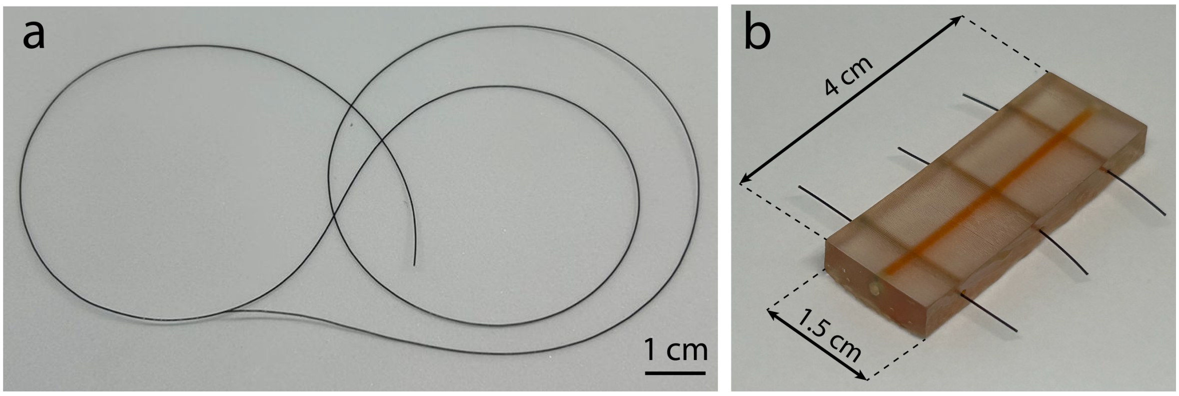

Figure S4. Digital pictures of (a) a MXene fiber used in this work and (b) an electronic tongue device composed of MXene-coated nylon fibers. Electrical contacts would be made at the ends of each fiber. The water was mixed with an orange dye to facilitate visualization of the analyzed solution inserted in the middle channel.

Table S1. Positions and integrated area of the peaks from the C 1s XPS spectra of Ti_3C_2, $Ti_3C_{1.75}N_{0.25}$, and $Ti_3C_{1.5}N_{0.5}$.

Peak	Binding Energy (eV)			Integrated Area (%)		
	Ti_3C_2	$Ti_3C_{1.75}N_{0.25}$	$Ti_3C_{1.5}N_{0.5}$	Ti_3C_2	$Ti_3C_{1.75}N_{0.25}$	$Ti_3C_{1.5}N_{0.5}$
**C-Ti**	281.95	281.91	281.92	33.77	29.48	11.00
**C-C**	284.55	284.96	284.64	51.45	50.90	70.75
**C-O**	286.15	286.30	285.97	9.24	13.47	9.02
**C=O**	288.85	288.87	288.30	4.53	6.16	9.23

Table S2. Positions and integrated area of the peaks from the Ti 2p XPS spectra of Ti_3C_2, $Ti_3C_{1.75}N_{0.25}$, and $Ti_3C_{1.5}N_{0.5}$ shown in Fig. 2b in the main text.

Peak	Binding Energy (eV)			Integrated Area (%)		
	Ti_3C_2	$Ti_3C_{1.75}N_{0.25}$	$Ti_3C_{1.5}N_{0.5}$	Ti_3C_2	$Ti_3C_{1.75}N_{0.25}$	$Ti_3C_{1.5}N_{0.5}$
**Ti 2p (3/2) - Peak I**	455.2	455.24	455.41	13.93	12.41	13.51
**Ti 2p (3/2) - Peak II**	456.14	456.29	456.48	29.71	23.01	25.5
**Ti 2p (3/2) - Peak III**	457.89	458.52	458.71	23.05	31.28	27.67
**Ti 2p (1/2) - Peak I**	461.26	461.24	461.38	6.96	6.2	6.75
**Ti 2p (1/2) - Peak II**	461.86	462.38	462.54	14.84	11.49	12.74
**Ti 2p (1/2) - Peak III**	462.96	464.27	464.46	11.51	15.62	13.82

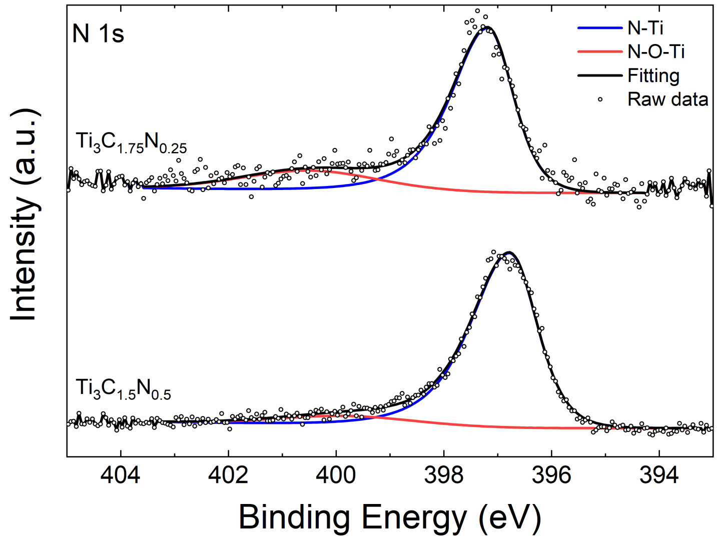

Figure S5. N 1s XPS high-resolution spectra of $Ti_3C_{1.75}N_{0.25}$ and $Ti_3C_{1.5}N_{0.5}$ MXenes.

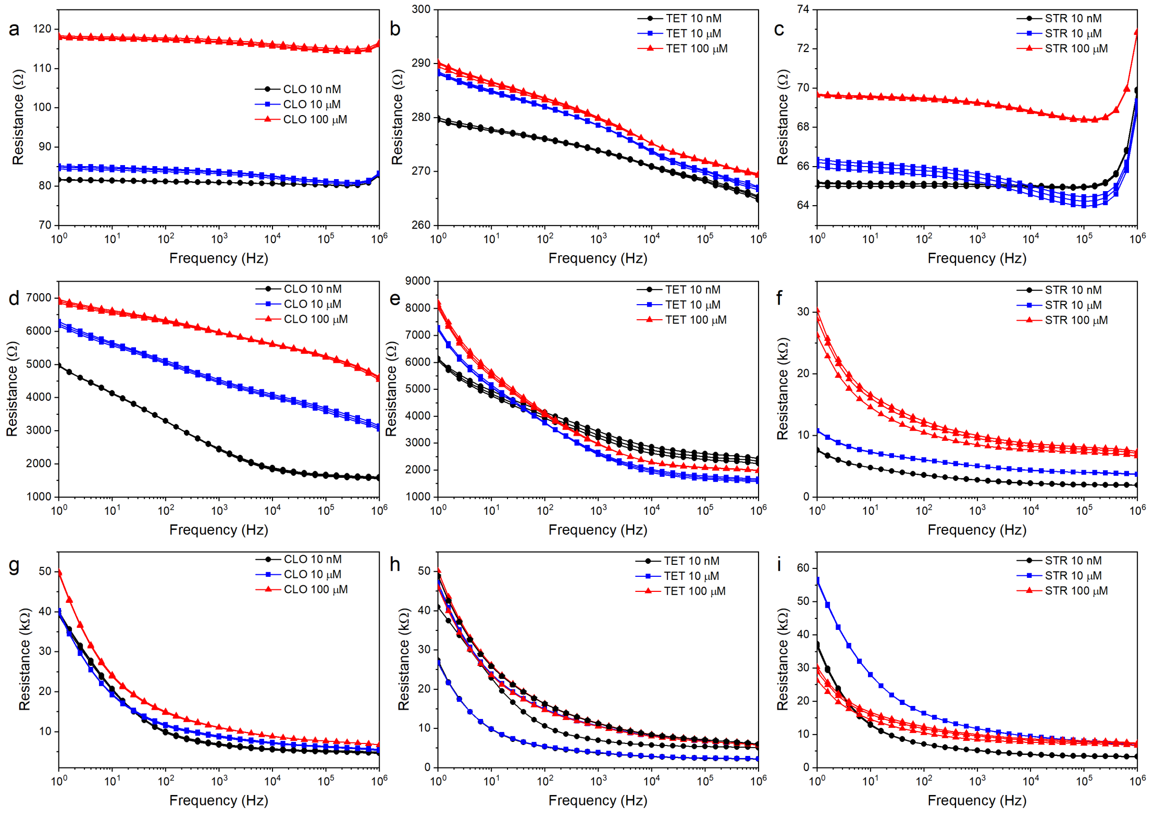

Figure S6. Electrical resistance versus frequency measured using MXene nylon fibers ((a-c) Ti_3C_2, (d-f) $Ti_3C_{1.75}N_{0.25}$, and (g-i) $Ti_3C_{1.5}N_{0.5}$) in PBS solutions spiked with cloxacillin (CLO), tetracycline (TET), and streptomycin (STR), respectively.

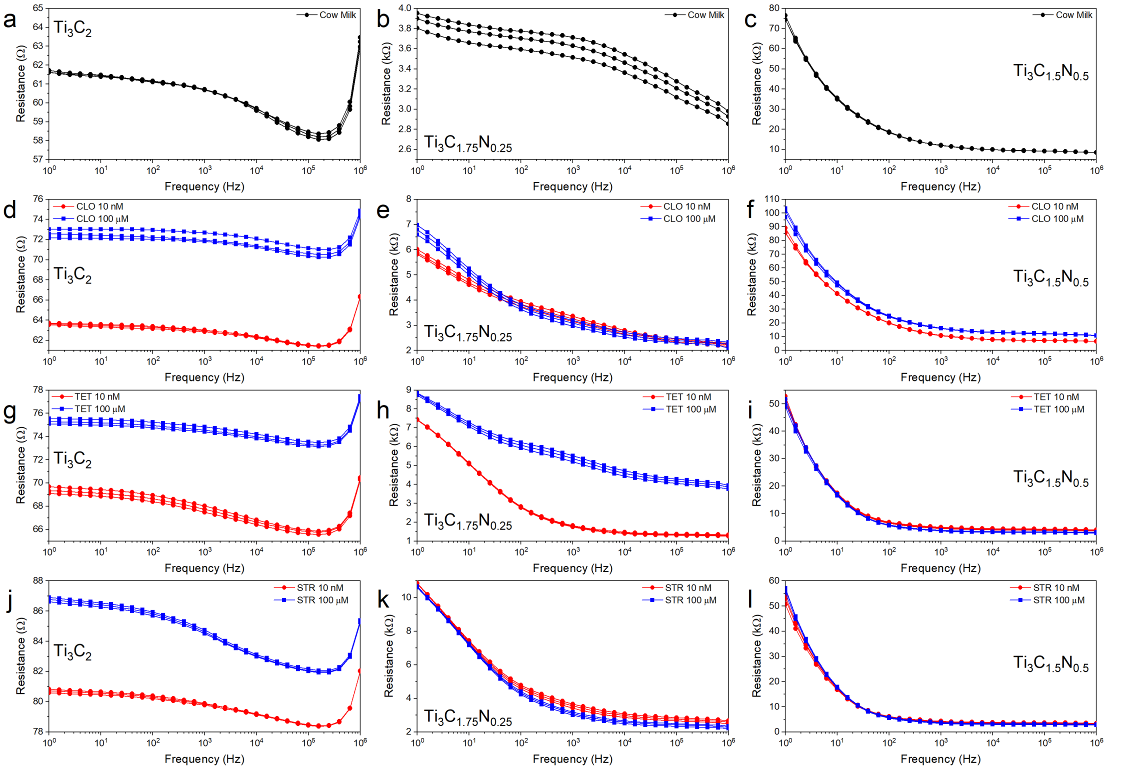

Figure S7. Electrical resistance versus frequency measured using three sensing units (Ti_3C_2, $Ti_3C_{1.75}N_{0.25}$, and $Ti_3C_{1.5}N_{0.5}$) of the e-tongue for (a-c) pure cow milk, and cow milk spiked with (d-f) cloxacillin (CLO), (g-i) tetracycline (TET), and (j-l) streptomycin (STR) at different concentrations.

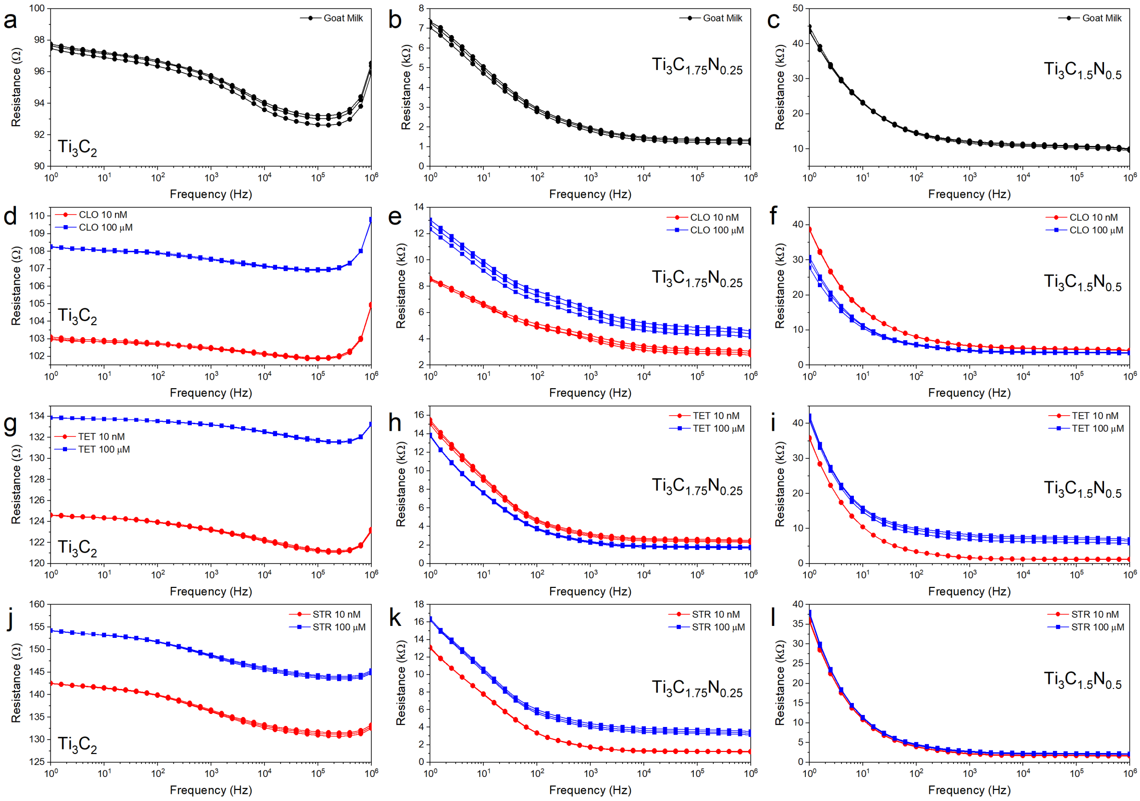

Figure S8. Electrical resistance versus frequency measured using three sensing units (Ti_3C_2, $Ti_3C_{1.75}N_{0.25}$, and $Ti_3C_{1.5}N_{0.5}$) of the e-tongue for (a-c) pure goat milk, and goat milk spiked with (d-f) cloxacillin (CLO), (g-i) tetracycline (TET), and (j-l) streptomycin (STR) at different concentrations.

.

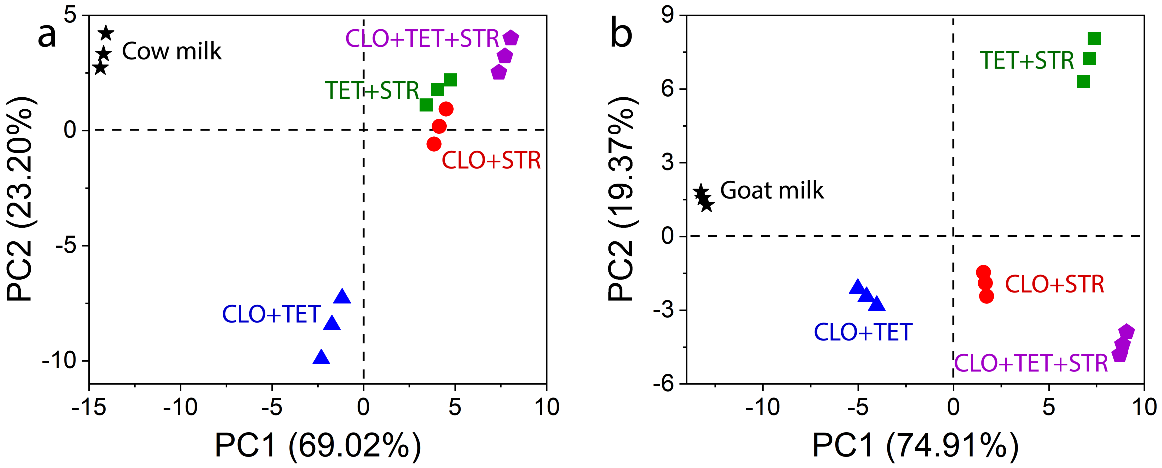

Figure S9. PCA plots for the electrical resistance measured by the MXene fibers-based e-tongue in the range from 1 MHz to 1 Hz for the analysis of solutions containing different combination of antibiotics (cloxacillin (CLO), tetracycline (TET), and streptomycin (STR)) at 100 µM in (a) cow and (b) goat milk samples.

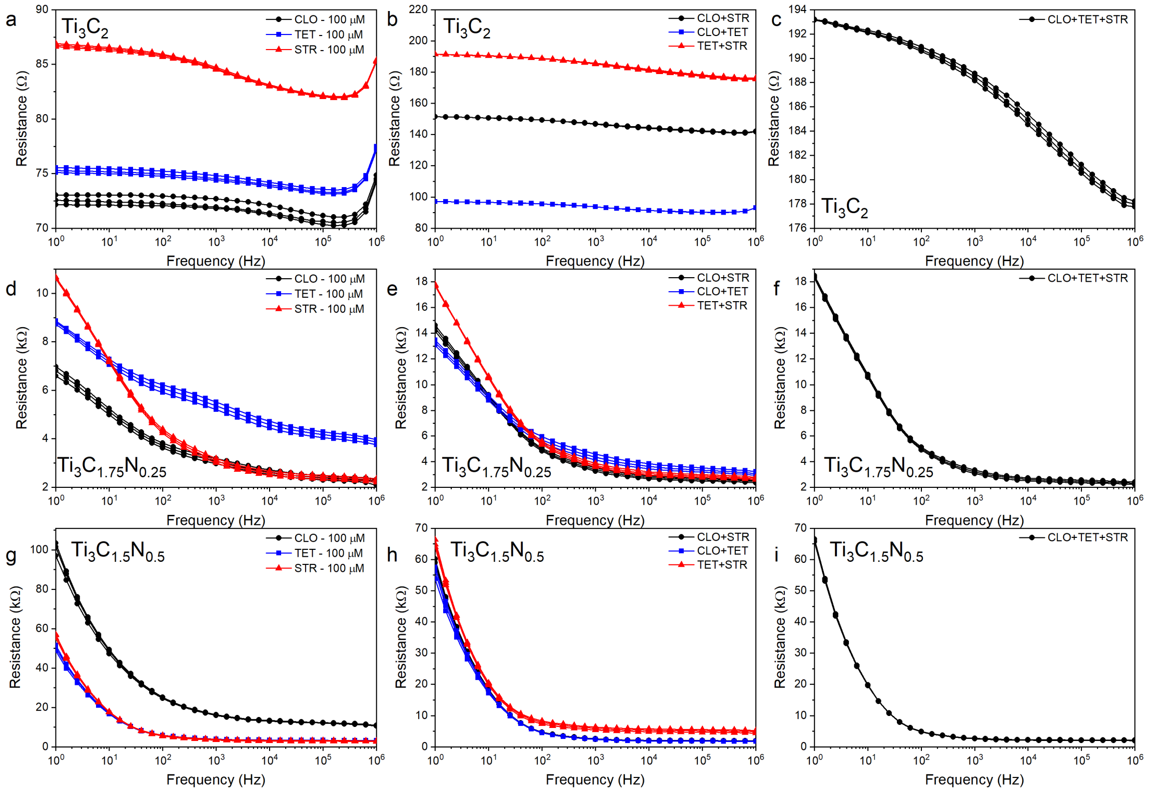

Figure S10. Electrical resistance versus frequency for (a-c) Ti_3C_2, (d-f) $Ti_3C_{1.75}N_{0.25}$, and (g-i) $Ti_3C_{1.5}N_{0.5}$ MXene nylon fibers in the analyses of antibiotic mixtures in cow milk.

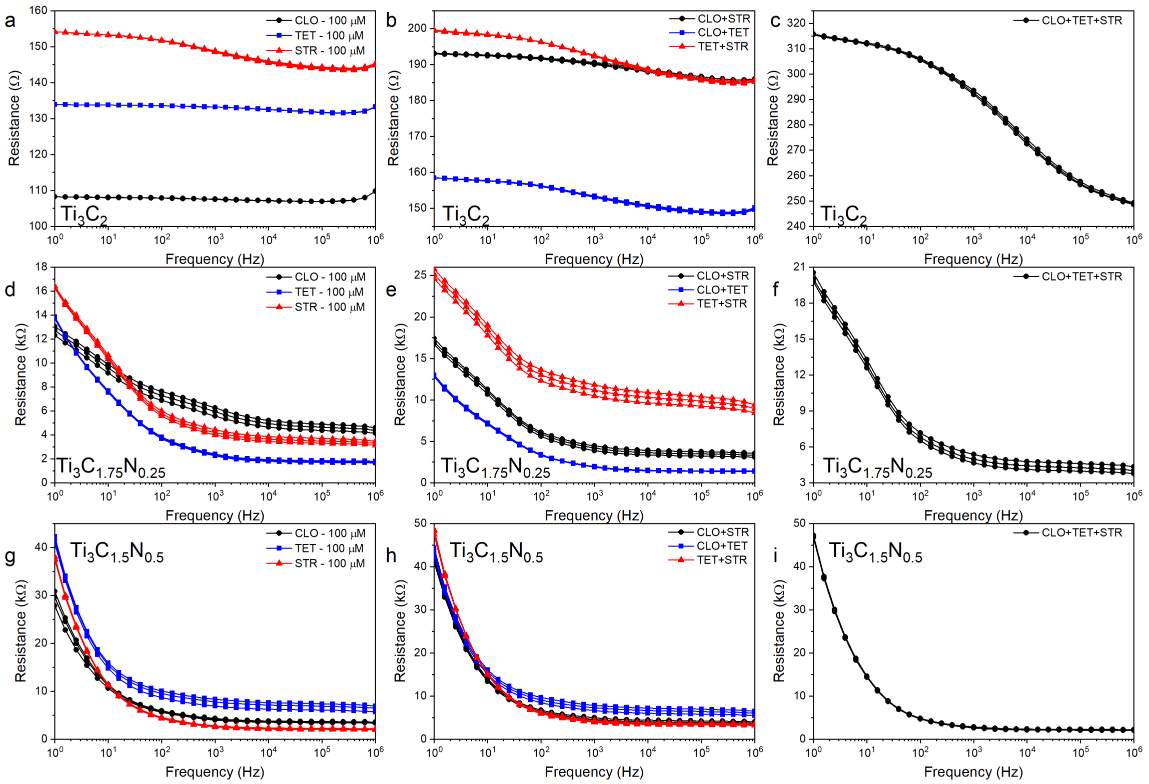

Figure S11. Electrical resistance versus frequency for (a-c) Ti_3C_2, (d-f) $Ti_3C_{1.75}N_{0.25}$, and (g-i) $Ti_3C_{1.5}N_{0.5}$ MXene nylon fibers in the analyses of antibiotic mixtures in goat milk.
